# A retrospective study assessing the factors associated with visual outcome in retinal vein occlusion patients after anti-VEGF therapy

**DOI:** 10.7717/peerj.12599

**Published:** 2021-12-06

**Authors:** Xiaoran Liu, Chi Xie, Yun Wang, Yue Xu, Shaojin Zhu, Yan Fang

**Affiliations:** 1 Medical College, Anhui University of Science and Technology, Huainan, China; 2 Department of Ophthalmology, the First Affiliated Hospital of Anhui University of Science and Technology, Huainan, China; 3 Department of Ophthalmology, the First People’s Hospital of Huainan, Huainan, China

**Keywords:** Retinal vein occlusion, Anti-VEGF therapy, Prognosis, Predictive factors

## Abstract

**Background:**

Retinal vein occlusion (RVO) is one of the most frequent retinal vascular diseases. In this study, we aimed to investigate the predictive factors of visual outcome for RVO patients who underwent anti-vascular endothelial growth factor (VEGF) therapy.

**Methods:**

RVO patients who underwent anti-VEGF treatment were recruited in this study from January 2018 to June 2020. Clinical data and optical coherence tomography (OCT) parameters were retrospectively reviewed. Best-corrected visual acuity (BCVA) was examined at baseline and after anti-VEGF therapy. Predictive factors associated with visual outcome were assessed by logistic regression model. Treatment-related adverse events were also recorded.

**Results:**

The average logMAR BCVA was 0.91 at baseline and 0.70 at final examination (*P* = 0.003). Among 75 patients, 41 experienced visual improvement were categorized as group A, the remaining 34 patients without improved vision were categorized as group B. Patients in group A demonstrated better visual outcomes, including decreased logMAR BCVA (average logMAR BCVA: 0.53 in group A *vs.* 0.91 in group B, *P* < 0.001) and central retinal thickness (CRT) (average CRT: 230.88 µm in group A *vs.* 404.97 µm in group B, *P* < 0.001) after anti-VEGF treatment. Multivariable analysis showed that injection frequency (odds ratio [OR], 2.623; 95% confidence interval [CI], [1.282–5.366]), hypertension (odds ratio [OR], 0.189; 95% CI [0.044–0.811]), hyperlipemia (odds ratio [OR], 0.195; 95% CI [0.040–0.941]) and external limiting membrane (ELM) disruption (odds ratio [OR], 0.148; 95% CI [0.032–0.691]) were all significantly associated with the visual outcome of RVO patients who underwent anti-VEGF treatment. In general, anti-VEGF therapy was feasible for all RVO patients, though the response to anti-VEGF was suboptimal in certain patients. Prognostic factors including injection frequency, hypertension, hyperlipemia and ELM disruption may all be useful to provide predictive information of visual outcome of RVO patients in response to anti-VEGF treatment.

## Introduction

Retinal vein occlusion (RVO) is a vascular disease of the retina which contributes to a major global burden of visual handicap (Song et al., 2019). The incidence of RVO in the general population is estimated at 5.2 per 1,000 persons (Laouri et al., 2011). RVO patients have an increased risk for hypertension, stroke and carotid artery disease (Chen et al., 2017; Zhou, Zhu & Wang, 2016). Thus, the incidence of RVO is likely to increase due to trends in global aging and cardiovascular disease (Song et al., 2019). It is estimated that baseline visual acuity is seriously damaged in RVO, ranging from 20/40 to less than 20/200 (Rogers et al., 2010). Additionally, the previous finding demonstrated that untreated RVO could result in macular edema (ME), retinal neovascularization as well as neovascular glaucoma (Petrella et al., 2012). The pathogenesis of RVO is characterized by the following features: intraretinal edema, retinal hemorrhages, dilated venules as well as vessel anastomoses (Rehak & Wiedemann, 2010).

A number of previous studies have demonstrated that vascular endothelial growth factor (VEGF) is an important factor contributing to the development of RVO. In RVO, the blood supply is decreased due to the blockage of venous circulation, leading to ischemia of the retina. It has been reported that VEGF expression is increased in ischemic retina and highly involved in the pathogenesis of RVO (Noma et al., 2005). One study found that VEGF triggered production of mitogen-activated protein (MAP), promoting endothelial cell proliferation, migration and angiogenesis (Ferrara, 2004). Intravitreal injections of anti-VEGF agents such as bevacizumab, ranibizumab and aflibercept have shown improved outcomes compared with the natural course of untreated RVO (Edington, Connolly & Chong, 2017; Moisseiev et al., 2014). However, in clinical practice we found that the response to anti-VEGF therapy was suboptimal in some patients. Furthermore, few previous studies have focused on the potential factors which affect the visual outcome of RVO patients who received anti-VEGF therapy. We sought to identify key prognostic factors of visual outcome of anti-VEGF therapy in RVO patients.

## Materials and Methods

### Study participants

**Figure 1:**
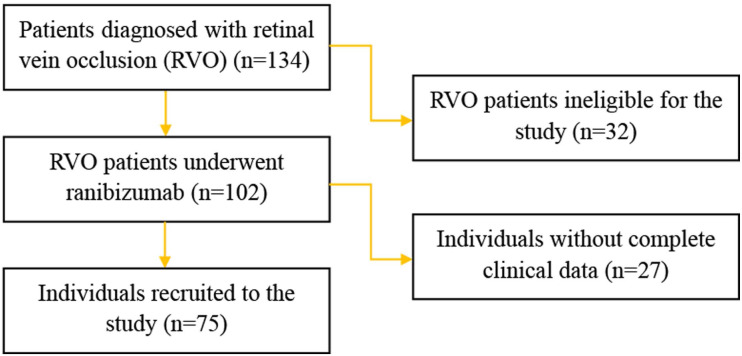
Flow chart of the study participants.

From January 2018 to June 2020 patients diagnosed with RVO who underwent anti-VEGF therapy were recruited. The study adhered to the tenets of the Declaration of Helsinki and was approved by the Ethics Committee of the First Affiliated Hospital of Anhui University of Science and Technology (No. 202013). The written informed consent was obtained from all patients recruited in this study. A flow chart detailing the selection of study participants was shown in Fig. 1. The inclusion criteria consisted of a diagnosis of RVO confirmed by funduscopic examination, fluorescein angiography and spectral domain optical coherence tomography (SD-OCT), all of which were routinely performed at baseline. Branch retinal vein occlusion (BRVO) was identified by the venous dilatation and tortuosity, superficial or deep hemorrhages and macular edema which involved only branches of the whole retinal venous system. Central retinal vein occlusion (CRVO) was characterized by the presence of the aforementioned, scattered across all four quadrants of the retinal venous system. The exclusion criteria were as follows: presence of other diseases affecting best-corrected visual acuity (BCVA), history of panretinal photocoagulation (PRP), cardiac event within prior 8 weeks, history of stroke, or allergy to any component of the anti-VEGF drug.

### Anti-VEGF treatment

Before injection, patients were treated with levofloxacin hydrochloride eye drops for 3 days. Topical anesthesia consisting of 0.4% oxybuprocaine was applied. Ranibizumab (Lucentis; 0.5 mg/0.05 mL, Novartis Pharma AG, Basel, Switzerland) was injected four mm posterior to the limbus, through the inferotemporal pars plana, with a 30-gauge needle. After initial anti-VEGF injection, pro re nata injections were administered in cases where central retinal thickness exceeded 250 µm or a decrease in BCVA was observed, according to the monthly follow-up (4 weeks after each injection).

### Clinical outcomes and assessment

All patients underwent a general health survey during their first hospital visit, including demographic and clinical profile such as age, gender, RVO type, time from onset to treatment, hypertension, hyperlipidemia and smoking habits, *etc*. A comprehensive ophthalmic examination was also carried out including best-corrected visual acuity (BCVA), which was determined as the logarithm of the minimum angle of resolution (Log MAR), slit-lamp examination, intraocular pressure measurement (IOP), funduscopic examination, fluorescein fundus angiography (FFA) and spectral domain optical coherence tomography (SD-OCT). The monthly follow-up examinations included BCVA, IOP, slit-lamp examination, funduscopic examination and SD-OCT. Patients were followed for 6 months. The total number of injections were also recorded for each patient. The primary efficacy outcome assessment was the mean change of BCVA from baseline to final examination. The secondary outcome was the mean change in central retinal thickness at baseline and post-operation. In the present study, we divided the patients into two groups according to BCVA improvement. Group A (good prognosis) was defined as improved BCVA after anti-VEGF treatment, and Group B (poor prognosis) was defined as no noticeable improvement in sight after anti-VEGF therapy. Adverse outcomes including vitreous hemorrhage, neovascular glaucoma, endophthalmitis and other adverse treatment related events were all recorded.

### Image grading

**Figure 2:**
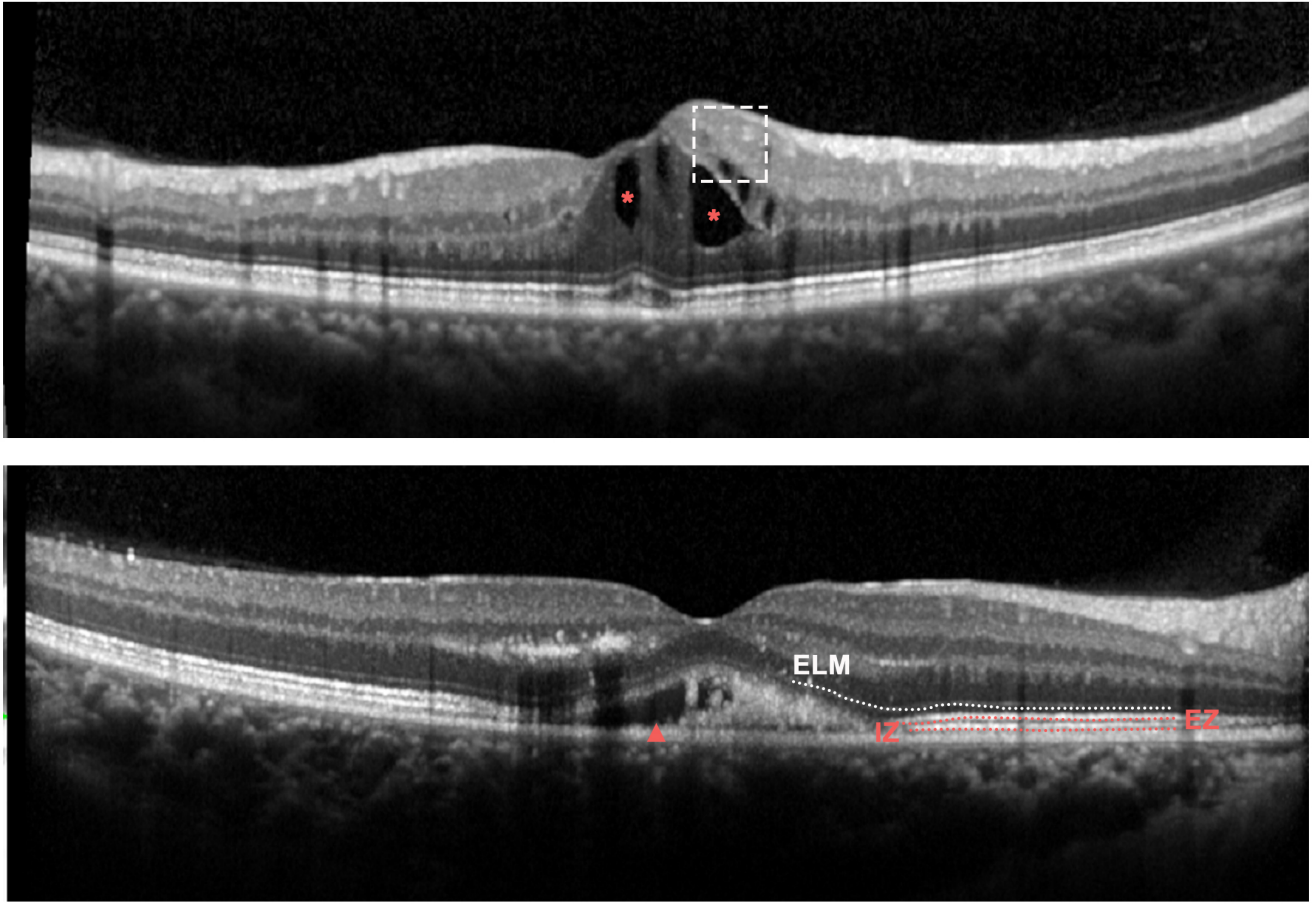
Representative OCT features at baseline in eyes with RVO. Upper panel: Spectral-domain OCT demonstrating DRIL (dashed line box) and IRF (red asterisk); Bottom panel: Spectral-domain OCT demonstrating SRF (red arrowhead), ELM disruption (white dotted line).

SD-OCT images were obtained with a confocal scanning laser ophthalmoscope (Heidelberg Engineering, Heidelberg, Germany). Horizontal and vertical scans through the fovea were recorded. Central retinal thickness (CRT) was defined as the average thickness of the central 1 mm circle of the retinal thickness map and detected by the caliper tool in the Heidelberg review software. Presence of the disorganization of retinal inner layers (DRIL), external limiting membrane (ELM) disruption, presence of the intraretinal fluid (IRF), presence of the subretinal fluid (SRF), ellipsoid zone (EZ) disruption as well as interdigitation zone (IZ) disruption were measured before anti-VEGF treatment (Fig. 2). DIRL was positively identified if either of the interfaces between the inner retinal layers (the ganglion cell layer and inner plexiform layer complex, inner nuclear layer and outer plexiform layer) could not be distinguished. The ELM, EZ and IZ lines were considered to be disrupted when they appeared discontinuous and had aberrant signal intensities compared to those of the peripheral macular area. All scans were assessed with double grading. To avoid potential segmentation errors by the OCT machine, specific manual corrections were performed by two experienced specialists when necessary.

### Statistical methods

For statistical analysis, the BCVA was analyzed on a logMAR scale. Clinical characteristics and OCT parameters were analyzed using frequency tabulations for categorical variables and summarized as means ± standard deviations (SD) for continuous variables. Student’s *t*-test and analysis of variance (ANOVA) were applied to compare between groups for continuous variables and Chi-square test was performed for categorical variables. Multivariate analysis was estimated with logistic regression to investigate potential associated factors, including age, gender, RVO type, time from onset to treatment, injection frequency, hypertension, hyperlipemia, smoking history and OCT parameters as candidate predictors for visual result using forward elimination.

## Results

### Patient characteristics

We screened 102 potentially eligible patients, 75 with unilateral RVO were finally enrolled in the study. Table 1 showed the baseline characteristics of study patients. Of 75 sets of eyes, 41 experienced visual gain after ranibizumab injection, and were classified as group A, the remaining patients were categorized into group B. Compared with patients in group A, those in group B were older and more likely to be men. The average number of injections was 2.63 in group A and 1.24 in group B. The median time from onset to treatment was 30 days for both groups. No significantly statistical difference was observed regarding age, gender, RVO type or time from onset to treatment between the two groups (Table 2), whereas injection frequency, comorbidities (including hypertension and hyperlipemia) and smoking were significantly different (Table 2). In terms of comorbidities, further analysis revealed that both therapeutic effect of hypertension and hyperlipemia were not statistically significantly different between group A and group B (*P* > 0.05). Hypertension control was compared between two groups for hypertensive patients, the number of patients with uncontrolled hypertension was 1 and 2 in group A and group B, respectively. For dyslipidemia patients, lipid level was tested at baseline to assess the attainment of lipid goal, of the 33 patients with hyperlipemia, nine subjects did not achieve the lipid goal (3 patients in group A and 6 patients in group B).

**Table 1:** Characteristics of included 75 patients with RVO.

Variables	Value
Age, (mean ± SD, years)	57.25 ± 11.94
Gender, male (n, %)	28, 37.33%
Type of RVO (n, %)	
CRVO	19, 25.33%
BRVO	56, 74.67%
Time from onset to treatment, (median)	30
Injections frequency (mean ± SD)	2 ± 1.36
Co-morbidities (n, %)	
Hypertension	33, 44
Hyperlipemia	33, 44%
Smoking (n, %)	41, 54.67%

**Notes.**

RVO Retinal vein occlusion CRVO Central retinal vein occlusion BRVO Branch retinal vein occlusion

**Table 2:** Characteristics of patients stratified according to BCVA.

Variables	Group A (*n* = 41)	Group B (*n* = 34)	*P* value
Age, (mean ± SD, years)	56.51 ± 12.01	58.15 ± 11.97	0.558
Gender, male (n, %)	13, 31.71%	15, 44.12%	0.269
Type of RVO, BRVO (n, %)	31, 75.61%	25, 73.53%	0.837
Time from onset to treatment, (median)	30	30	0.970
Injections frequency (mean ± SD)	2.63 ± 1.48	1.24 ± 0.65	<0.01
Co-morbidities (n)			
Hypertension	10	23	<0.01
Hyperlipemia	12	21	0.005
Smoking (n)	17	24	0.012

**Notes.**

BCVA best-corrected visual acuity RVO Retinal vein occlusion BRVO Branch retinal vein occlusion

### Baseline OCT parameters

On baseline SD-OCT data, mean CRT was 544.09 µm. More than 85% of eyes demonstrated IRF and 62% of eyes showed SRF. The proportion scans with the presence of the DRIL and ELM disruption were 32 (42.6%) and 42 (56%), respectively. In addition, EZ disruption and IZ disruption were detected in 39 (52%) and 42 (56%) eyes, respectively. The initial SD-OCT findings for each group were shown in Table 3. There was no statistical difference detected in average pre-injection CRT between group A and group B (567.61 µm in group A *vs.* 515.74 µm in group B, *P* = 0.340). More presence of the DRIL and ELM disruption were observed in group B than those in group A. Meanwhile, the presence of the IRF, the presence of the SRF, EZ disruption and IZ disruption were not significantly different between two groups.

**Table 3:** Ocular parameters of patients stratified according to BCVA.

Variables	Group A (*n* = 41)	Group B (*n* = 34)	*P* value
DRIL (n)	12	20	0.010
ELM disruption (n)	16	26	0.001
IRF (n)	34	30	0.750
SRF (n)	23	24	0.197
EZ (n)	19	20	0.281
IZ (n)	19	23	0.064
CRT at baseline (mean ± SD, µm)	567.61 ± 236.82	515.74 ± 228.26	0.340

**Notes.**

BCVA best-corrected visual acuity DRIL Disorganization of the retinal inner layers ELM External limiting membrane IRF Intraretinal fluid SRF Subretinal fluid EZ Ellipsoid zone IZ Interdigitation zone CRT Central retinal thickness

### Clinical outcomes

**Figure 3:**
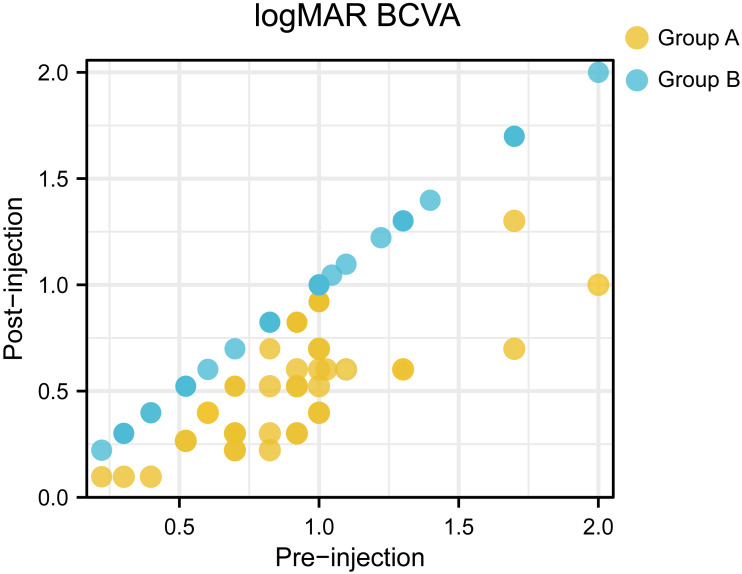
A scatter plot showing the logMAR BCVA at baseline and final examination for each patient.

**Figure 4:**
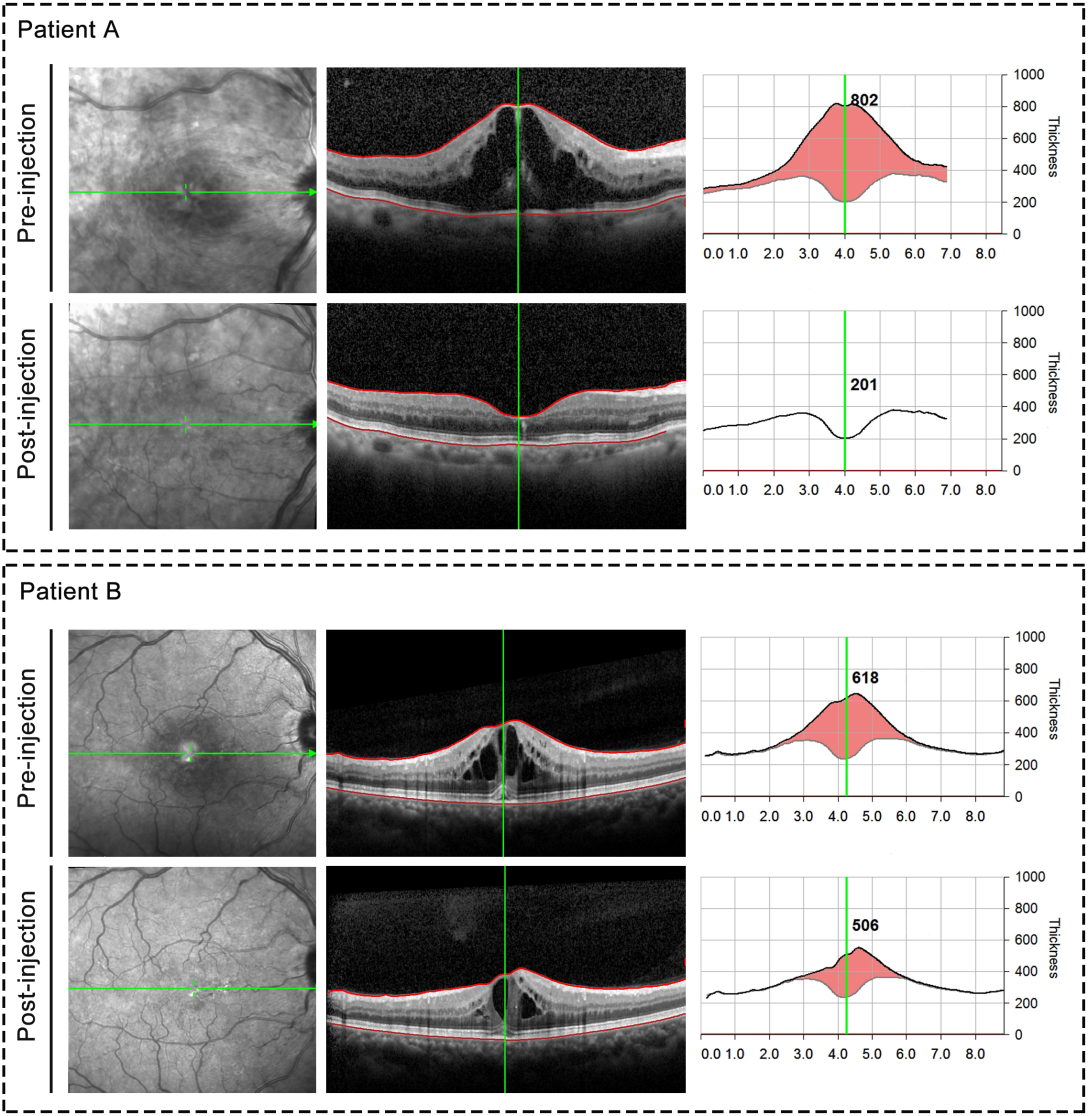
Representative OCT images taken before and after ranibizumab injection. Upper panel: Representative case from group A; Bottom panel: Representative case from group B.

Of the 75 patients, the mean initial logMAR BCVA was 0.91 ± 0.40, after anti-VEGF injection, the mean logMAR BCVA was 0.70 ± 0.42. There existed significant difference between initial logMAR BCVA and post-injection logMAR BCVA (*P* = 0.003). The initial logMAR BCVA was 0.90 ± 0.34 in group A and 0.91 ± 0.46 in group B, with no significant (*P* = 0.924) between-group difference at baseline. The BCVA was significant improved after anti-VEGF treatment in group A, whereas group B showed no increase in BCVA. Figure 3 presented a scatter plot showing the logMAR BCVA at baseline and final examination for each patient. Central retinal thickness as an indicator of macular edema also demonstrated significant differences before and after treatment (544.09 µm before treatment *vs* 309.80 µm after treatment, *P* < 0.001). Moreover, group A demonstrated significant differences in average CRT pre- and post-injection (567.61 µm before injection and 230.88 µm after injection, *P* < 0.001). And compared with those in group B, patients in group A showed significantly decreased central retinal thickness after anti-VEGF injection (average CRT after injection: 230.88 µm in group A *vs.* 404.97 µm in group B, *P* < 0.001). Representative OCT images taken before and after ranibizumab injection were shown in Fig. 4. Finally, two cases of ocular hypertension following the administration of ranibizumab were obtained, and the symptoms relieved after additional treatment.

### Prognostic factors for visual outcome

**Figure 5:**
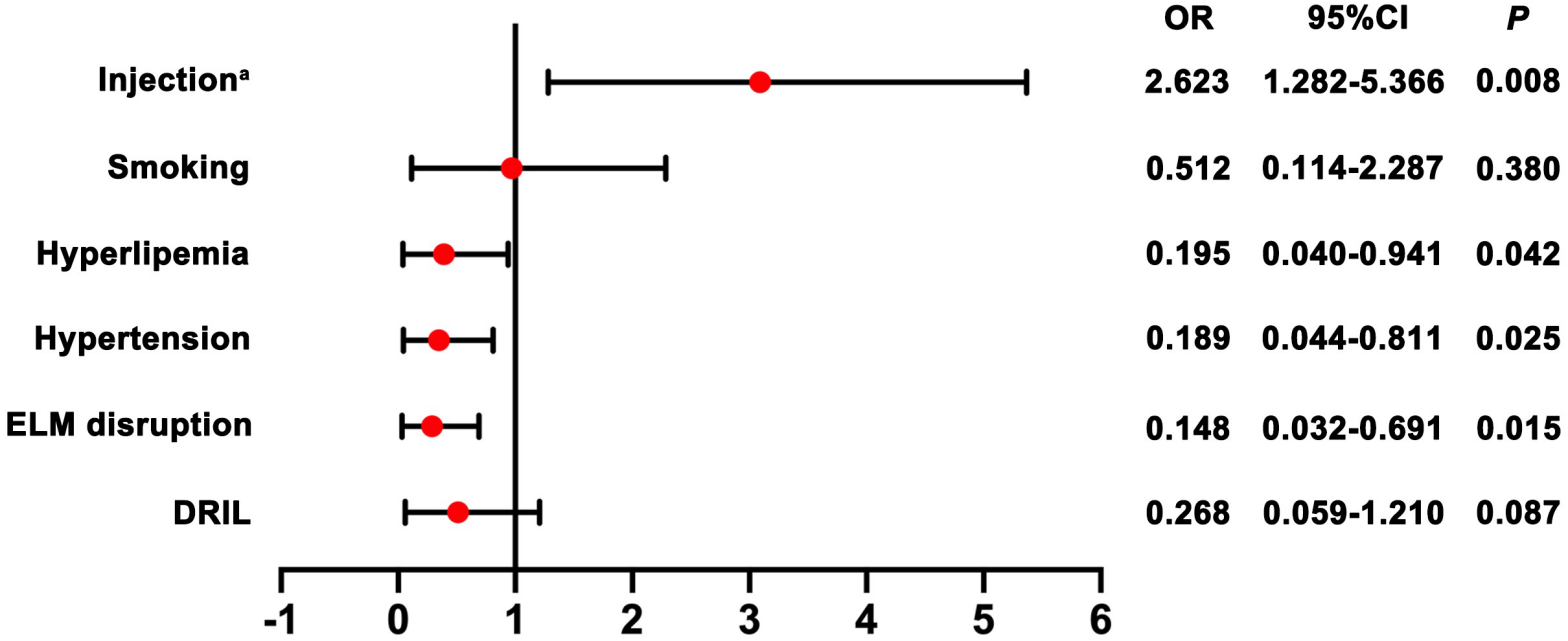
Forest plots with results of the effect of potential prognostic factors for visual outcome. Note: a indicates injection frequency.

To identify predictors for prognosis after injection, we selected candidate predictive factors based on previous retrospective studies and our experience, including age, gender, type of RVO, time from onset to treatment, injection frequency, hypertension, hyperlipemia, smoking history as well as OCT parameters. A univariate analysis showed statistical significance for all these potential factors tested. As shown in Tables 2 and 3, univariate analysis showed injection frequency, hypertension, hyperlipemia, smoking, presence of DRIL and ELM disruption were significant prognostic factors. Multivariate analysis further confirmed that increased injection frequency (OR = 2.623; 95% CI [1.282–5.366]), absence of hypertension (OR = 0.189; 95% CI [0.044–0.811]), absence of hyperlipemia (OR = 0.195; 95% CI [0.040–0.941]) and intact ELM (OR = 0.148; 95% CI [0.032–0.691]) were all independent prognostic factors for improved visual outcome (Fig. 5).

## Discussion

Retinal vein occlusion ranks as the second leading cause of retinal vascular blindness all around the world. Previous study has demonstrated that VEGF is highly involved in the development of macular edema and the size of the nonperfused areas in eyes affected by RVO (Noma et al., 2005). D’Amico et al. (2017) reported that exogenous VEGF led to pathologic alteration in eyes such as microaneurysm formation, intraretinal hemorrhages and venous beading. Thus anti-VEGF therapy has been promoted for the management of RVO. Anti-VEGF agents include bevacizumab, ranibizumab and aflibercept. Several studies have investigated the effect of anti-VEGF agents against RVO. Ivanovska Adjievska et al. (2017) reported that intravitreal bevacizumab provides improvement in visual acuity and reduction of macular edema in a high percentage of treated eyes after 1 year. The results from the RETAIN study additionally described good long-term results in BRVO patients treated with ranibizumab (Campochiaro et al., 2014). In line with previous studies, we found that anti-VEGF therapy was beneficial for RVO patients in terms of visual improvement. Our study revealed that logMAR BCVA decreased from a baseline of 0.91 to 0.70 after ranibizumab treatment, which represented a significant improvement in vision. Additionally, average CRT was significantly decreased after ranibizumab injection, corroborating the findings of previous reports, which showed ranibizumab to be effective for visual improvement.

Although inhibition of the VEGF levels in RVO is beneficial for the relief of macular edema and suppression of fibrovascular membrane formation, recent clinical studies have reported that some patients show a poor response to anti-VEGF treatment (Larsen et al., 2018; Nagasato et al., 2020). Our findings also revealed that more than one third of patients exhibited no visual improvement after anti-VEGF treatment. To the best of our knowledge, the factors associated with visual outcome after anti-VEGF treatment have not been fully elucidated. In this study, we retrospectively investigated potential predictive factors for the visual outcome of RVO patients who underwent anti-VEGF treatment. Our findings showed that injection frequency of ranibizumab, hypertension, hyperlipemia and ELM disruption were independent factors for visual outcome of RVO patients with anti-VEGF treatment. Wecker et al. (2019) reported that frequency of anti-VEGF injections had a significant impact on visual outcome in the treatment of neovascular age-related macular degeneration. Another study reported that RVO patients receiving multiple injections of anti-VEGF agents acquired long-term visual acuity development (Maggio et al., 2020). This association was consistent with our study where more frequent injections generate better visual outcome. Apart from injection frequency, comorbidities were associated with prognosis of RVO as well. Previous findings have confirmed a strong link between hypertension and RVO (Wong & Mitchell, 2007). And a number of previous studies have also confirmed that hyperlipemia is one of the risk factors contributing to developing RVO (Kim et al., 2019; O’Mahoney, Wong & Ray, 2008). In our study, more than one third of patients experienced hypertension and hyperlipemia, with the univariate analysis and final multivariate analysis, our data demonstrated that hypertension and hyperlipemia impaired the effect of anti-VEGF agents and predicted the poor prognosis of RVO. Additionally, several studies have revealed OCT parameters are related to visual acuity in vascular eye diseases (Li et al., 2020; Sun et al., 2014). For example, a relationship between the pre-treatment status of the ELM and post-treatment visual outcome has been described for eye diseases (Liu et al., 2018; Theodossiadis et al., 2011). ELM is a barrier within the retina and comprises a region of zonular adherence between Muller cells and the photoreceptor layer, which is essential for the maintenance of normal vision (Ota et al., 2010). Wolf-Schnurrbusch et al. (2011) found that intact ELM was associated with better visual outcome after anti-VEGF treatment in patients with macular edema secondary to CRVO. Another study revealed central subfield thickness, ELM and EZ status could be useful in clinical practice as predictors for visual outcome of anti-VEGF treatment (Tang et al., 2020). We believed that those hypotheses could be partially confirmed by our results in which ELM disruption was independent factor for visual outcome in RVO patients with anti-VEGF treatment. Besides, some studies investigated the role of DRIL in predicting RVO prognosis. For instance, Mimouni et al. (2017) reported a decreasing trend of DRIL during the follow-up period of patients who had RVO with anti-VEGF treatment. We found that DRIL was significantly associated with poor visual acuity in univariate analysis. However, in the final multivariate model we noted that only ELM disruption, as oct parameter, was statistically significant predictive factor. The present study has some limitations due to its retrospective nature and limited sample size. As such, large prospective controlled trials are needed to verify these findings. In addition, with the development of diagnostic and therapeutic approaches, more techniques such as optical coherence tomography angiography (OCTA) should be evaluated to provide better decision-making for RVO patients.

## Conclusion

In summary, we confirmed that anti-VEGF therapy was reliable for most RVO patients and that prognostic factors might be of clinical use to provide predictive information for visual outcome in response to anti-VEGF treatment. However, further studies with prospective and case-controlled designs are needed to verify these conclusions.

## Supplemental Information

10.7717/peerj.12599/supp-1 Supplemental Information 1 Raw data

## Additional Information and Declarations

### Data Availability

The following information was supplied regarding data availability: The raw measurements are available in the Supplementary File.
